# Programmable Valve With Virtual off Function Is Useful for Chronic Subdural Hematoma After Ventriculoperitoneal Shunt Surgery

**DOI:** 10.7759/cureus.75508

**Published:** 2024-12-10

**Authors:** Kento Tsuburaya, Takashi Matsumori, Masashi Uchida, Hiroshi Takasuna, Hidetoshi Murata

**Affiliations:** 1 Neurosurgery, St. Marianna University School of Medicine, Kawasaki, JPN; 2 Neurosurgery, Graduate School of Medicine, Yokohama City University, Yokohama, JPN

**Keywords:** chronic subdural hematoma (csdh), codman certas plus programmable valve, overdrainage, ventriculoperitoneal (vp) shunt, vp shunt surgery

## Abstract

Over-drainage after a ventriculoperitoneal (VP) shunt can often lead to chronic subdural hematoma; however, the treatment is unclear. Hematoma drainage is performed after physically stopping the shunt function, such as by ligating or removing the shunt system. However, shunt reconstruction is required after the subdural hematoma improves. This study investigated the usefulness of a programmable valve with virtual off-function for chronic subdural hematoma after VP shunt surgery. We installed a programmable valve with a virtual off-function for chronic subdural hematoma after VP shunt surgery. A programmable valve with a virtual off-function is a valve system that can stop and restart the shunt function at will. After the subdural hematoma has improved, shunt function can be resumed, and pressure can be set to an optimal level. Installing a programmable valve with a virtual off-function is useful as a new treatment method to address over-drainage complications while preserving shunt function.

## Introduction

Chronic subdural hematoma is often caused by ventriculoperitoneal (VP) shunt over-drainage. Chronic subdural hematomas occur in 5-10% of cases [1,2]. The most common treatment for improving subdural hematoma after a VP shunt is temporarily stopping the shunt function and removing the hematoma. Shunt ligation is often a reliable method to stop shunt function [1,2]. However, if the shunt is ligated, it must be promptly reconstructed for hydrocephalus after the subdural hematoma improves. However, in recent years, programmable valves with a virtual off function have become available, allowing conventional pressure changes and shunt on/off function, which facilitates dealing with over-drainage complications such as subdural hematoma after VP shunt surgery. Herein, we describe the usefulness of a programmable valve with a virtual off-function that can turn the shunt function on and off for chronic subdural hematoma after VP shunting.

## Case presentation

**Figure 1:**
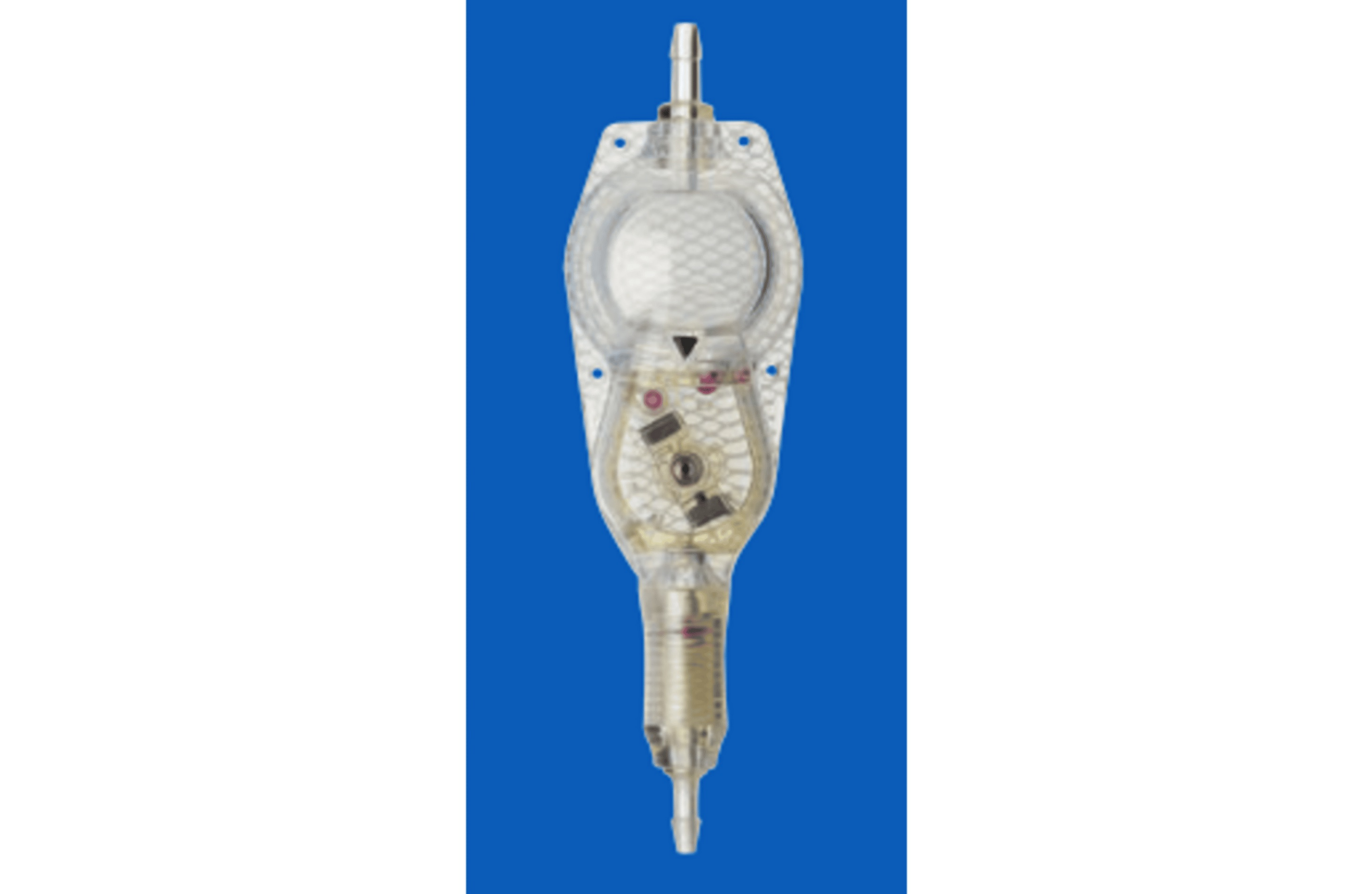
Exterior photo of programable valve with virtual off function An exterior photo of the programable valve with a virtual off function (Codman CERTAS plus®, Integra LifeSciences, NJ, USA). This image is used with permission from the publisher (©Integra Japan K.K. 2020 HYDRO-001 1209869-3).

As a treatment for chronic subdural hematoma with over-drainage after a VP shunt, if the shunt valve has a virtual off function, we turn off the shunt function (virtual off) and remove the hematoma. If the shunt valve does not have a stop function, we switch to a valve with a virtual off function or construct an additional valve and remove the hematoma after the shunt function is virtual off. We used the programable valve with virtual off function (Codman CERTAS Plus®, Integra LifeSciences, NJ, USA, launched in the United States in 2015 and in Japan in 2016) (Figure 1) to also allow hydrocephalus treatment after hematoma removal.

**Figure 2:**
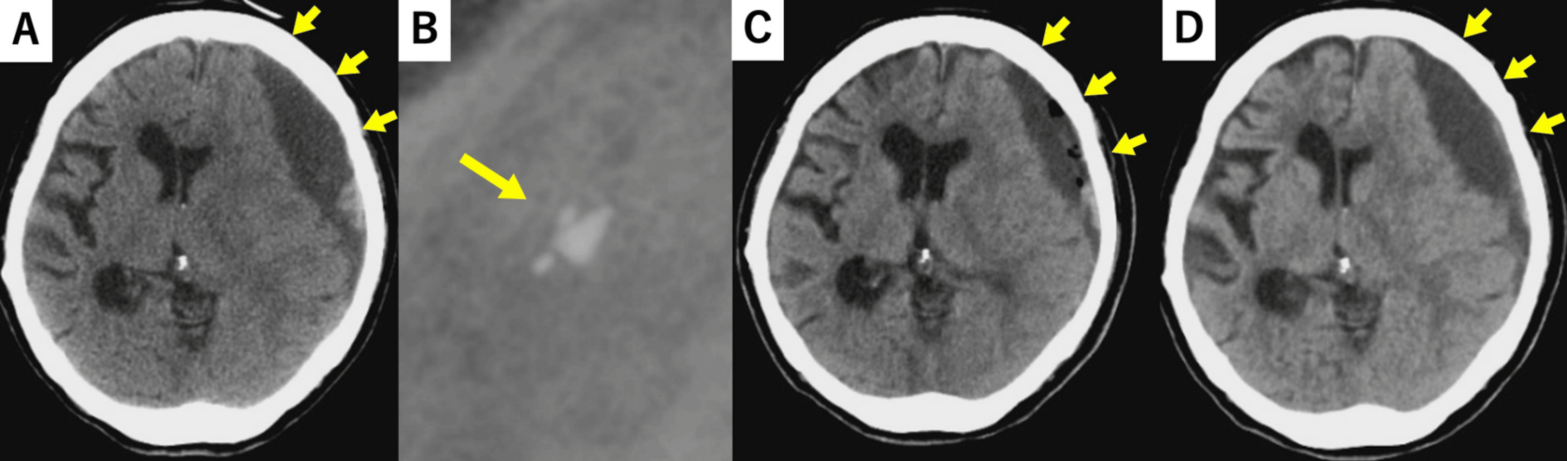
A: Plain CT axial image of the head on admission; B: Plain radiograph of the head on admission; C: CT immediately after burr hole hematoma removal; D: CT on the fourth day after the initial surgery A: Left chronic subdural hematoma with midline shift was observed. B: The shape of the valve marker revealed it as a fixed valve (NovusTM Valve, Natas, USA). C: Temporary decrease in hematoma observed. D: The hematoma had re-enlarged, and the midline shift had worsened.

We present a case study using a programable valve with a virtual off function. An 87-year-old woman had undergone VP shunt surgery via right anterior horn puncture at another hospital ten years prior. During hospitalization at the previous hospital, she presented with impaired consciousness and right hemiplegia. Head CT revealed a left chronic subdural hematoma with midline shift, and she was transferred to our hospital for surgery (Figure 2A). Radiography revealed that the shunt valve was a fixed-pressure valve (Novus Valve, Natus, WI, USA)（Figure 2B). Over-drainage was not evident because the VP shunt had been in use for a long time. Therefore, hematoma drainage with a burr hole was performed under local anesthesia on the day of admission. Although the hematoma temporarily decreased after the operation (Figure 2C), a CT on the fourth day of admission showed that the hematoma had enlarged to the same size as it had been on admission (Figure 2D). The disturbance of consciousness and right hemiplegia did not improve.

**Figure 3:**
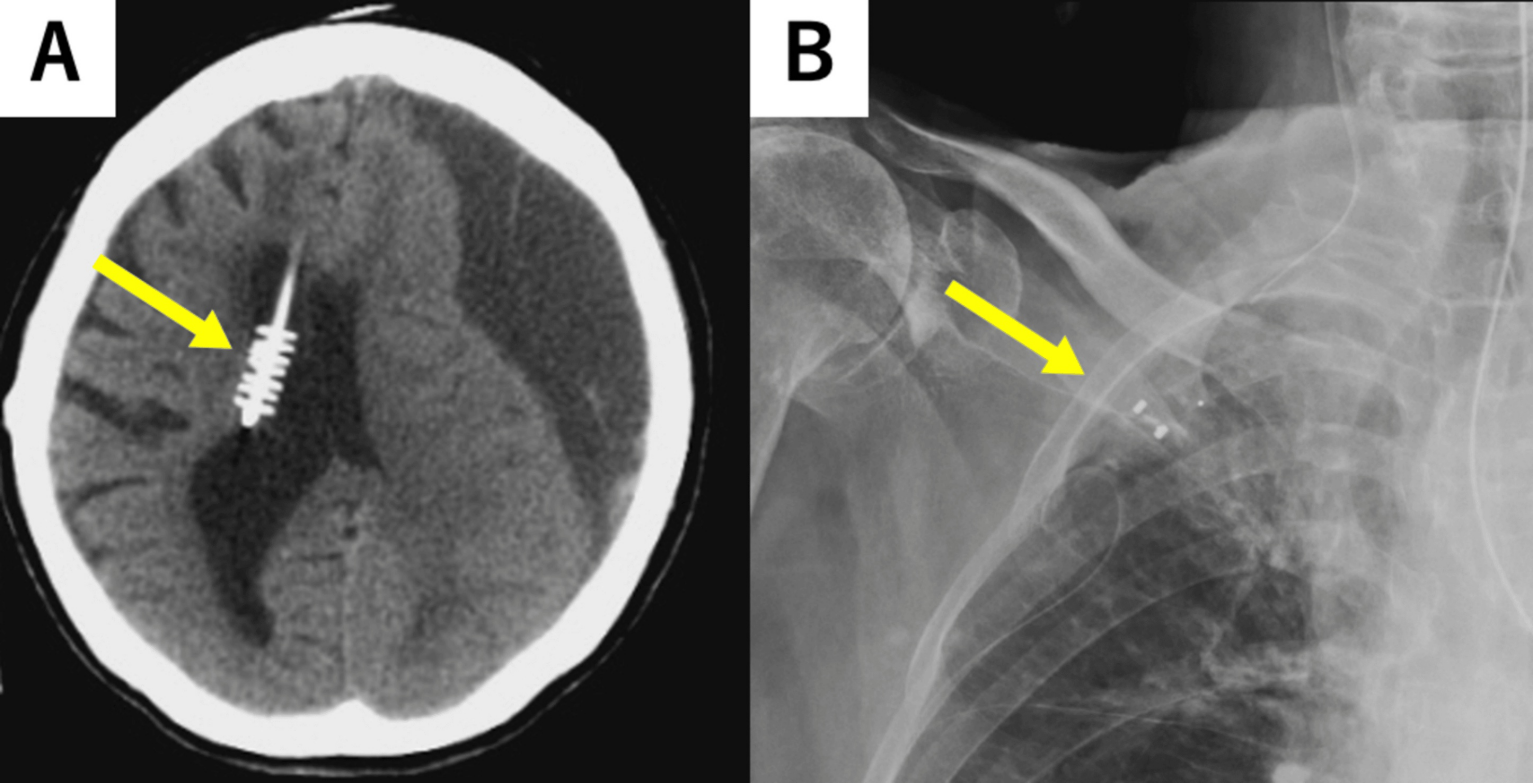
A: CT scan on admission; B: Plain chest radiograph after adding a valve A: A shunt was placed from the right anterior horn, and the tip of the tube had a flange shape. B: A programmable valve with a virtual off-function was added to the right anterior chest.

**Figure 4:**
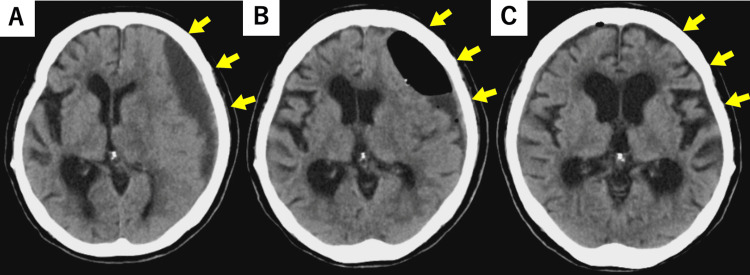
A: CT on the eighth day of admission (third day after the addition of the new valve); B: 10th day of admission (fifth day after the addition of the new valve); C: CT on the 25th day of admission (15th day after hematoma cleaning) A: The ventricle had enlarged over time, but the hematoma remained. B: CT immediately after hematoma cleaning C: The hematoma had almost disappeared.

Eliminating the over-drainage associated with the VP shunt was deemed necessary. The valve pressure was fixed, the pressure could not be adjusted, and the tip of the ventricular tube was flange-shaped, hindering its removal (Figure 3A). The fixed pressure valve was installed at another hospital 10 years ago, so detailed information was unavailable. It was also difficult to determine the pressure from the X-ray. Considering the risk of recurrence of hydrocephalus after subdural hematoma improvement, we decided to add a programable valve with virtual off function (Codman CERTAS Plus®) (Figure 1) to the existing VP shunt instead of ligating the shunt tube. On the fifth day after admission, we added the valve with a virtual offset to the right anterior chest under local anesthesia (Figure 3B) and set it off. We performed the additional surgery using a clean technique in the operating room, administered prophylactic antibiotics, and performed skin disinfection and draping. We also performed frequent wound irrigation during surgery. Although the ventricles gradually expanded postoperatively, the reduction in the hematoma was insufficient (Figure 4A). Therefore, we performed hematoma drainage again under local anesthesia on the 10th day of admission (Figure 4B). We placed a subdural drain and confirmed improvement in consciousness disturbance, right hemiplegia, and reduction in hematoma. The drain was removed on the 14th day of admission. We normally do not leave the drain in place for a long time, but we judged there to be a high possibility of recurrence and decided to remove it once the hematoma had sufficiently reduced. Even after the drain was removed, the hematoma decreased over time. CT performed on the 25th day after admission revealed that the hematoma had almost disappeared (Figure 4C). Although the ventricles were enlarged, no hydrocephalus symptoms were observed. We planned to release the virtual offsetting if hydrocephalus symptoms recurred; however, as no symptoms occurred, the patient was transferred to a rehabilitation hospital on the 32nd day of admission, with the virtual offset still on.

## Discussion

Patients with VP shunts often develop chronic subdural hematomas. The cause is cerebrospinal fluid over-drainage associated with shunt surgery. In this study, a programable valve with a virtual off function has been useful as a new method to deal with this problem.

Although there is no established treatment for chronic subdural hematoma after a VP shunt, it is essential to prevent over-drainage. If the chronic subdural hematoma is not massive in volume, the hematoma may disappear simply by improving over-drainage. Ma et al. reported a case in which chronic subdural hematoma after valve damage due to trauma was successfully treated by changing the valve to a programable valve [3]. Serarslan et al. reported that fixed valves in 10 patients were changed to variable pressure valves [4]. Switching to a programmable valve is one solution to over-drainage complications. Still, a randomized trial between programmable and fixed valves revealed no significant difference in the occurrence of subdural hygroma [5].

A more reliable treatment for subdural hematoma is to stop the shunt function [1,2]. Among these, the ligation of the shunt tube is common [1,2]. Zemack et al. reported 30 cases of chronic subdural hematoma after VP shunt placement; 10 patients underwent surgery, and 7 underwent ligation [1]. Sundtrom et al. reported that 90% of patients with fixed valves required some surgical procedure [2]. Carmel et al. reported a case in which a patient with a fixed valve showed improvement after shunt removal and drainage [5].

However, if the shunt is ligated or removed, hydrocephalus may recur after the subdural hematoma decreases in size. Moreover, cases have also been reported wherein the shunt ligation was removed because the condition deteriorated rapidly after ligation [5]. Shunt reconstruction is required if the shunt system needs to be ligated or removed. Furthermore, if a chronic subdural hematoma recurs, surgery to stop the shunt system must be repeated.

Using a programable valve with a virtual off function can solve this problem. With the virtual offsetting, the pressure is 400mmH2O or more, and we believe it has the same effect as ligation or removal. The shunt function can be stopped as a valve system, and the shunt function can be restarted immediately after the hematoma has improved. The intraventricular pressure can be changed to the optimal pressure depending on the ventricular pressure, and even if the subdural hematoma recurs, the shunt system can be deactivated again without physical damage to the shunt system (virtual off). In addition, compared to the button type that was previously used, this magnetic valve is thought to have a lower risk of failure. In this way, it is easy to manage over-drainage complications such as subdural hematoma. Installing a programmable valve with a virtual off-function is a useful new method to treat over-drainage complications, such as chronic subdural hematoma after shunting. In the future, accumulating more cases and conducting further investigations will be necessary.

## Conclusions

Over-drainage after shunt surgery can sometimes cause a chronic subdural hematoma, but no clear treatment has been established. Shunt reconstruction is required if the shunt system needs to be ligated or removed. Installing a programmable valve with a virtual off-function for chronic subdural hematoma associated with over-drainage after a VP shunt is a new method that can deal with over-drainage while preserving shunt function.
